# Effect of hypoxia-inducible factor-1α on transcription of survivin in non-small cell lung cancer

**DOI:** 10.1186/1756-9966-28-29

**Published:** 2009-02-26

**Authors:** Yu-Qing Chen, Cheng-Ling Zhao, Wei Li

**Affiliations:** 1 Department of Respiration, First Affiliated Hospital of Bengbu Medical College, 227 Chang Huai Road, Bengbu, Anhui 233004, PR China

## Abstract

**Background:**

Survivin is a structurally and functionally unique member of the inhibitor of apoptosis protein (IAP) family. It plays an important role, not only in regulating mitosis but also in inhibiting apoptosis. The current literature contains few reports on the transcriptional regulation of survivin expression in lung cancer.

**Methods:**

In this study, we investigated the effect of hypoxia-inducible factor-1α (HIF-1α) on the transcriptional activity of the survivin promoter in non-small cell lung cancer (NSCLC). Immunohistochemical staining was used to detect the expression of survivin and HIF-1α in the lung tissue of 120 patients with non-small cell lung cancer (NSCLC) and 40 patients with benign pulmonary disease. We also performed experiments with the lung adenocarcinoma cell line A549 cells, which were cultured under hypoxic conditions. The expression of survivin and HIF-1α was detected by real-time RT-PCR and Western blotting. In the survivin promoter the putative binding-site for HIF-1α, is -19 bp~-16 bp upstream of TSS. We performed site-directed mutagenesis of this binding site, and used luciferase reporter plasmids to determine the relative activity of the survivin promoter in A549 cells. We also studied the effect of HIF-1α on the expression of survivin by dsRNA targeting of HIF-1α mRNA.

**Results:**

HIF-1α (58.33%) and survivin (81.60%) were both over-expressed in NSCLC and their expressions correlated with one another. They were also expressed in A549 cells under normal and hypoxic conditions, with a significant increase under hypoxic conditions. Site directed mutagenesis of the putative binding site for HIF-1α in the survivin promoter significantly decreased the activity of the survivin promoter in A549 cells. Inhibition of HIF-1α by RNAi decreased the expression of survivin in A549 cell lines.

**Conclusion:**

Our results indicate that the binding of HIF-1α to the survivin promoter increases transcription of the survivin gene. Thus, HIF-1α is an important transcriptional regulator of survivin expression

## Background

Survivin is a structurally and functionally unique member of the inhibitor of apoptosis protein (IAP) family. It plays an important role not only in regulating mitosis but also in inhibiting apoptosis [1,2]. Moreover, it is highly expressed in almost all types of human tumors and fetal tissues but barely detectable in normal adult tissues [3,4]. High levels of survivin expression have been associated with tumor progression and angiogenesis, resistance to radiation and drug treatments, and poor survival rates in cancer patients [5,6]. Different approaches aimed to target survivin, including small interfering RNAs [7], dominant negative mutants [8], antisense oligonucleotides [2], ribozymes [9,10], and triplex DNA formation [11], have been used for cancer treatment. However, none of these studies focus on transcriptional inhibition of survivin as a potential approach for cancer treatment. Due to the multiple functions of survivin, it seems that transcriptional inhibition of survivin could be an important mechanism to inhibit survivin expression for cancer treatment [12,13].

Much effort has been made to explore the mechanisms by which survivin transcription is regulated. A previous report indicates that the survivin gene promoter is TATA-less and contains GC-rich sequences. Additionally, the Sp1 transcription factor induces survivin expression in HeLa cells [14]. The core promoter of survivin contains multiple CACCC or GGGTG motifs for binding of Sp1-like proteins and Kruppel-like factors (Sp/KLF) [3]. For example, KLF5, a member of Sp/KLF family, was found to be a stimulator for survivin expression in Acute Lymphoblastic Leukemia [15]. However, there are few reports related to the transcriptional regulation of survivin in lung cancer and the precise molecular mechanism of survivin transcriptional regulation remains unclear.

Poor oxygenation (hypoxia), owing to an inadequate blood supply, is a common feature of most solid human tumors and is associated with increased malignancy, resistance to therapy and distant metastasis [16]. Hypoxia inducible factor-1α (HIF-1α), a member of basic helix-loop-helix-PAS protein family [17,18], is usually increased under hypoxic conditions, and can activate transcription of many genes that are critical for cellular function under hypoxic conditions [17]. Previous studies have found that down-regulation of HIF-1α could significantly decrease the levels of survivin expression in BxPc-3 pancreatic cancer cells [19] and breast cancer cells [20]. These data indicated that HIF-1α regulates expression of survivin. However, there are very few studies on mechanisms of survivin expression regulated by HIF-1α. Recently, Peng et al demonstrated that HIF-1α could directly bind to the survivin promoter, which strongly suggests that survivin gene expression is indeed mediated by f HIF-1α under normoxic conditions [20]. Nonetheless, there are still no related reports in lung cancer.

In the present study, we examined the expression of HIF-1α and survivin in tumor tissue from patients with non-small cell lung cancer (NSCLC) as well as in a NSCLC cell line derived from human lung adenocarcinoma (A549). We found that HIF-1α and survivin were widely expressed in both A549 cells and fresh NSCLC tissue samples and that HIF-1α expression was consistently associated with high levels of survivin expression in the lung cancer samples. By analyzing the survivin promoter activity, we further found that HIF-1α was a transcriptional activator of the survivin gene.

## Methods

### Tissue Specimens

Tissue samples were obtained from patients with a pathological diagnosis of NSCLC as determined by two pathologists. Patients were operated on in Department of Thoracic Surgery, the First Affiliated Hospital, Bengbu Medical College during the period from March 2005 to November 2007. There were 160 patients who signed informed consent forms to participate in the study, among them 120 NSCLC patients, 40 patients with benign pulmonary disease (36 chronic inflammation, and 1 case each of pulmonary haemorrhage, pulmonary fibrosis, inflammatory pseudotumor, and hamartoma). Patients received no chemotherapy or radiotherapy before operation. The patients characteristics: male 94, female 26; age 42–76, average 61 years old; histology: squamous cancer 100 cases, adenocarcinoma 20 cases; well-differentiated cancer 45 cases, moderately differentiated cancer 46 cases and poorly differentiated cancer 29 cases; TNM staging: 43 cases in stage I-II and 77 cases in stage III according to 1997 revised version of lung cancer staging standard by International Union Against Cancer (UICC). All of the patients had complete follow-up data and received conventional post-surgery chemotherapy. The principle committee of the First Affiliated Hospital of Bengbu Medical College had authorized this research.

### Reagents

Goat anti-human survivin monoclonal antibody and anti-human HIF-1α monoclonal antibody were purchased from Santa Cruz (Santa Cruz, CA, USA). Lipofectamine™ 2000 and Trizol were purchased from Invitrogen (Carlsbad, CA, USA). pGEM-T-EASY vector, pGL3-basic vector, pGL3-control vector, pRL-Tk vector and the Dual-Luciferase^® ^Reporter Assay System were purchased from Promega (Madison, WI, USA). Universal Gene DNA Extraction Kit Ver.3.0, PrimeScript™ RT-PCR KIT, Agarose Gel DNA Purification kit2.0, Minibest Plasmid Purification kit 2.0, TaKaRa MutanBEST Kit, PrimerSTARTM HS DNA Polymerase, SYBR PrimeScript™ RT-PCR Kit were purchased from Takara BioTechnology Co., Ltd (Dalian, China). The dATP was purchased from Fermentas (Burlington, Canada). Primers were synthesized by Sangon Biological Engineering Technology & Services Co., Ltd (Shanghai, China).

### Cell line and culture

Human lung adenocarcinoma cell line A549 was maintained in Dulbecco's modified Eagle's medium (DMEM), supplemented with 10% fetal bovine serum (Invitrogen,), 100 units/ml of penicillin and 100 mg/ml of streptomycin (Invitrogen) in a humid atmosphere incubator with 5% CO_2 _at 37°C. To study the expression of survivin induced by hypoxia, A549 cells were incubated in hypoxic condition (1% O2, 5% CO2 and 94% N2) for 24 h.

### Immunohistochemistry

Immunohistochemical staining using the streptavidin peroxidase method (S-P method) was performed on 4-μm sections of paraffin-embedded specimens to detect expression of survivin and HIF-1α protein in NSCLC and benign lung disease tissues. In brief, after deparaffinization and hydration, the slides were treated with endogenous peroxidase in 0.3% H_2_O_2 _for 30 min, after which the sections were blocked for 2 hrs at room temperature with 1.5% blocking serum in phosphate-buffered saline (PBS). Sections were then incubated with anti-Survivin antibody (1:200 dilution) or anti-HIF-1α antibody (1:200 dilution) at 4°C overnight., followed by washing in PBS, and incubation with secondary anti-mouse biotinylated antibody (1 : 2000) in PBS for 30 min at 37°C. Antibody binding was detected using the streptavidin-biotin-peroxidase complex/HRP, Code K0377 (Dako), with 3,3 diaminobenzidine for 3 min as a chromogenic substrate. Finally, the slides were lightly counterstained with hematoxylin. As a negative control, duplicate sections were immunostained without exposure to primary antibodies. The results were observed under a light microscope.

### PCR-based Site Directed Mutagenesis of survivin promoter

Genomic DNA of A549 cells was extracted with Universal gene DNA extraction kit ver.3.0 according to the manufacturer's instructions. Survivin core promoter 230 bp (-203 ~ +27 bp) was amplified by PCR using primers with sequences selected from the survivin core promoter sequence; (Forward: 5'-ATC GAC GCG TTC TTT GAA AGC AGT CGA GGG GGC-3', Reverse: 5'-CCC AAG CTT TCT GGC GGT TAA TGG CGC GCC-3',). The cycling parameters were 95°C for 10s as a pre-denature step, followed by 40 cycles of 95°C for 5s, and 55°C for 30s, 72°C for 10 min. PCR products were purified, a polyadenylated by T4 DNA ligase, and then cloned to T-vector, named pGEM-T-EASY-sur230 bp. The template for site-directed mutagenesis was pGEM-T-EASY-sur230 bp. The forward and reverse primers (Forward: 5'-AGC GCT CCC GAC ATG CCC CGC GGC-3', Reverse: 5'-GCC CTCTTA GGC GGT CCA C-3') were used for PCR amplification. The cycling parameters were 30 cycles of 95°C for 10s, 60°C for 5s, 72°C for 30s. The linear product was self ligated after a blunting kination reaction; the product was named pGEM-T-EASY-sur229 bp and confirmed by sequencing.

### Construction of survivin promoter-luciferase reporter vectors, and transfection into A549 cells

The mutant, and normal constructs were removed from pGL3-basic by restriction endonuclease *Mlu I/Hind III*. The reporter vectors were constructed by T4 DNA ligase, named pGL3-SVP-229-luc (mutant) and pGL3-SVP-230-luc (normal). A549 cells were plated onto 6-well plates one day prior to transfection. Following confirmation of 70%–80% confluence, the cells were transfected with pGL3-Basic without promoter (negative control), pGL3-SVP-229-luc (mutant plasmid), and pGL3-SVP-230-luc (normal plasmid). For cell transfection, A549 cells were transiently transfected with 2 μg plasmids and 0.2. g internal control plasmid pRL-TK by using Lipofectamine 2000™ reagent according to the manufacturer's instructions.

### Luciferase reporter gene expression detection

Thirty hours after transfection, cells were harvested and lysed with 1 × lysis buffer (Promega), and then 20 μl of cell extract was assayed for luciferase activity using the Dual-Luciferase assay kit (Promega) according to the manufacture's instructions. The relative level of reporter gene expression was expressed as the ratio of firefly luciferase activity to *Renilla *luciferase (LU/RL).

### RNA interference

A double strand siRNA oligonucleotide targeting HIF-1α (sense: 5-CUGAUGAC CAGCAACUUGAdTdT-3, antisense: 5-UCAAGUUGCUGGUCAU CAGdTdT-3) was designed based on the reference [21] and synthesized by Shanghai Genepharma Co. Ltd. (China). A pair of negative control siRNA were also designed with sequences different from siRNA-HIF-1α and not homologous to any sequences found in gene bank (sense: 5-AGUUCAACGACCAGUAGUCdTdT-3, antisense: 5-GACUACUGGUCGUUGA dTdT-3). For transfection, cells were plated onto 10 cm^2 ^cell culture dishes and grown to 30–50% confluence before transfection. 50 μl of Oligofectamine transfection reagent per dish (Invitrogen) was added, and the cells were incubated at room temperature for 20 min. The cells were then rinsed with Opti-Mem I to remove any residual serum. The siRNA duplexes were diluted to a final concentration of 20 nM in Opti-Mem I (Invitrogen). Cells were incubated with the oligonucleotide duplexes in serum-free conditions for 4 h at 37°C. Serum was then added back to the culture, and cells were incubated in normoxic or hypoxic condition for an additional 48 h.

### Real Time Reverse Transcription-PCR

Total RNAs were isolated using Trizol reagent (Invitrogen) according to the manufacturer's instruction. Twenty-five nanogram total RNA per sample was reverse transcribed by using the Reverse Transcription Reaction Kit (Takara Code: DRR061S) according to the manufacturer's instructions. Quantitative real-time PCR was performed analyzed on the Applied Biosystems 7300 Real-Time PCR System to determine the relative amounts of survivin, HIF-1α and GAPDH (internal control) mRNAs expressed. The SYBR Green Supermix was used for all real-time PCR reactions. The primers used in this study were: forward: 5'-AGCCA GACGATCAT GCAG CTACTA-3 ', and reverse: 5'-TGTGGTAAT CCACTTT CATC CAT TG-3 ' for HIF-1α PCR product (167 bp); forward: 5'-AGGTCATCTCGGCTGTTCCTG-3', and reverse: 5'-TCATCCTCACTGCGGCT GTC-3', for survivin PCR product (147 bp); and forward: 5'-GGTCTCCTCTGAC TTCAACA-3', and reverse: 5'-AGCCAAATTC GTTGTCATAC-3' for GAPDH PCR product (116 bp). The quantitative real-time PCR PCR parameters were 95°C for 10s as a pre-denature step, followed by 40 PCR cycles of 95°C for 5 s and 60°C for 30 s, and 72°C for 10 min. Data presented in this study was collected at 60°C applying a threshold of 0.002 and normalized to GAPDH using the default RQ ddCt study software.

### Western Blot Analysis

After treatment, cells were washed two times with ice-cold PBS and then lysed by Cell Lysis Solution (DSL, USA). Cell lysates were incubated for 20 min at -20°C, and then centrifuged at 13,000 g for 20 min at 4°C. Supernatants were collected and protein concentration was determined by the Bradford method. Fifty microgram of protein from each sample was subjected to SDS-PAGE. After electrophoresis, proteins were transferred from the gel to polyvinylidene difluoride (PVDF) membranes (Millipore MA, USA). After blocking in a solution of 5% non-fat dry milk diluted in TBS, the membranes were washed, and incubated with primary antibodies [goat anti-survivin (1/200), rat anti HIF-1α (1/200), or rat anti-β-actin (1/800)] for 3 h at room temperature. After washing, the membranes were incubated with the appropriate horseradish peroxidase-labelled secondary antibody (1/2000) for 1 h. Blots were developed using a chemiluminescent detection system (ECL, Amersham Biosciences, Buckinghamshire, UK).

### Statistical analyses

The samples were analyzed by Q test, analysis of variance and Chi-square tests to determine whether there were significant differences between individual groups. The correlation of survivin and HIF-lα protein in NSCLS was analyzed by Spearman correlation analysis. All the tests were performed using SPSS 11.5, and *p *< 0.05 was considered significant.

## Results

### Expression of survivin and HIF-1α in NSCLC and benign lung disease tissues

**Figure 1:**
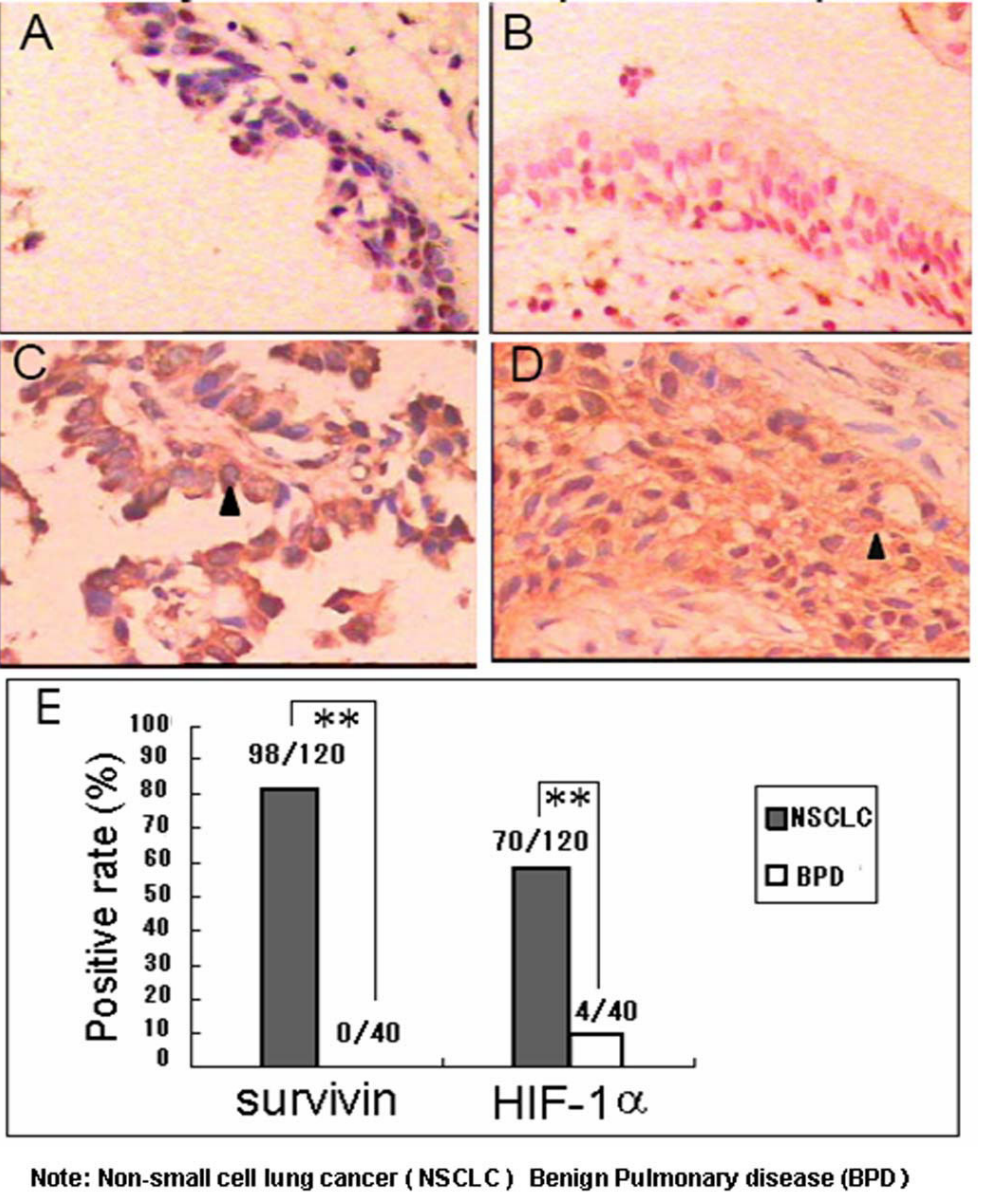
**Expression of survivin and HIF-1α in NSCLC and benign lung disease tissues**. Survivin and HIF-lα protein were detected and localised within paraffin-embedded human lung tissue using immunohistochemistry. A and B represent the negative expression of survivin protein and HIF-1α protein, respectively, in benign lung disease tissues. C and D represent the positive expression (arrow) of survivin protein and HIF-1α protein, respectively, in NSCLC,. E: The graph shows the statistical results. 81.60% (98/120) of lung cancer tissue samples were positive for survivin staining, and 58.33% (70/120_) of lung cancer tissue samples were positive for HIF-1α staining. ** *p *< 0.01.

Survivin and HIF-lα proteins were detected and localised in paraffin-embedded human lung tissue sections using immunohistochemistry. Survivin was predominantly expressed in the cytosol of the tumour cells with some nuclear staining (Fig. 1C). Survivin was exclusively expressed in lung cancer tissue (Fig. 1C, E, 81.60%,) and not in benign lung disease tissue (Fig. 1A, E, 18.4%). The specificity of survivin protein in lung cancer was 100%. HIF-lα was found primarily in the cytosol of lung cancer cells, with some nuclear staining (Fig. 1D). Positive rate of HIF-lα in lung cancer tissue samples was 58.33% (70/120), higher than that in tissue samples from benign lung disease (10%, 4/40) (Fig. 1B, E, *p *< 0. 01). The expression of survivin or HIF-1α in NSCLC was not correlated with age or sex, but with differentiation grade, lymph node metastasis and disease stages (Table 1). Spearman correlation analysis showed a correlation between the expression of survivin and the expression of HIF-1α in (*r*_s _= 0.255, *p *= 0.005) (Table 1).

**Table 1:** The correlation of survivin and HIF-1α expression with clinical pathology in NSCLC.

		HIF-1α protein			Survivin protein		
Clinical Pathology	N	positive	positive rate (%)	*p*	positive	positive rate (%)	*p*
sex							
male	94	56	59.6	>0.05	79	84.0	>0.05
female	26	14	53.8		19	73.1	
age							
≤60	67	41	61.2	>0.05	52	77.6	>0.05
>60	53	29	54.7		46	86.8	
degree of differentiation							
high	45	19	42.2	<0.01	31	68.9	<0.01
moderate	46	29	63.0		39	84.8	
low or undifferentiation	29	22	75.9		28	96.6	
clinical stage							
I~II	43	18	41.9	<0.01	29	72.1	<0.01
III	77	52	67.5		69	87.0	
lymph nodes metastasis							
yes	73	49	67.1	<0.01	66	90.4	<0.01
no	47	21	44.7		32	68.1	
Survivin Positive**	98	63	90(63/70)				= 0.005

Note: ** : *r*_s _= 0.255, *p *= 0.005.

#### Hypoxia induces expression of HIF-1α and survivin

**Figure 2:**
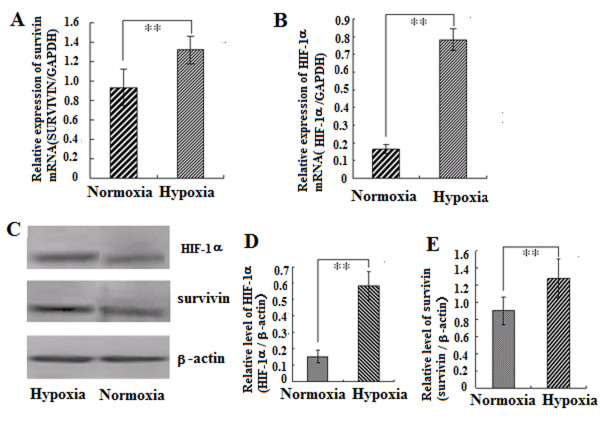
**Hypoxia induces expression of HIF-1α and survivin**. A549 cells were cultured in 10% FBS medium under hypoxic or normoxic conditions for 24h. The relative levels of survivin (A) and HIF-1α (B) to GAPDH mRNA were determined by quantitative real time, reverse transcription-PCR. C: The expression of survivin and HIF-1α protein in A549 cells following HIF-1α-siRNA treatment as detected by Western blot analysis. D: The graph shows the statistical results of relative expression level of survivin and HIF-1α to β-actin protein. Data are given as means ± SD, n = 3, ** p < 0.01.

When A549 cells were incubated in hypoxic conditions for 24 h, the expression of HIF-1α (2B, C, D) and survivin (2A, C, D) were detected by quantitative real time, reverse transcription-PCR (2A, B) and western blot (2 C, D). As shown in Fig 2, the expression of survivin and HIF-1α was increased significantly in hypoxia as compared to normoxia (p < 0.01).

#### Site directed mutagenesis of HIF-1α binding site on the survivin promoter decreases transcription activity of the survivin promoter

**Figure 3:**
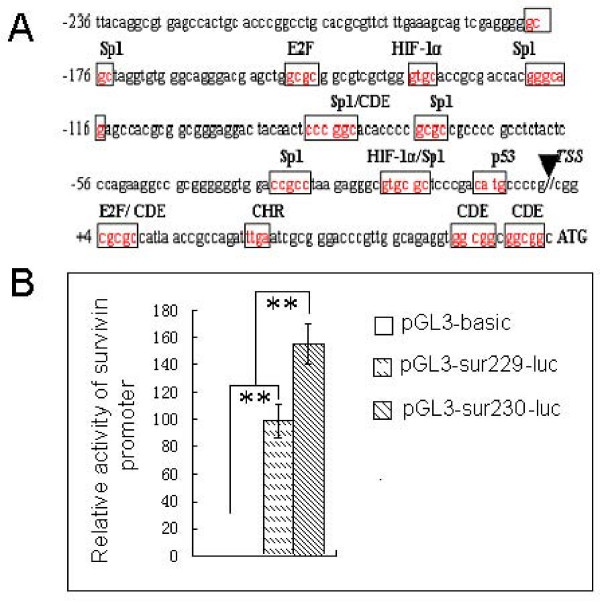
**Site directed mutagenesis of the HIF-1α binding site on the survivin promoter decreases transcription activity of the survivin promoter**. A: Nucleotide sequence of the survivin promoter. The putative binding sites for transcription factor are boxed. The GTGC sequence in -19 ~ -16 bp of survivin promoter was changed to AGC by mutation. B: A549 cells were transfected with pGL3-Basic without promoter (negative control), pGL3-SVP-229-luc (mutant plasmid), and pGL3-SVP-230-luc (normal plasmid). The relative activity of survivin promoter was analyzed by luciferase assay. The graph shows the statistical results. Data are given as means ± SD, *n *= 3, ** *p *< 0.01.

To determine whether the binding-site of HIF-lα can affect the transcription of survivin in A549 cells, the GTGC sequence in -19 ~ -16 bp of survivin promoter (Fig. 2A) was changed to AGC by site-directed mutagenesis, and the relative activity of the normal and mutated survivin promoter were detected by luciferase activity assay. As shown in Fig. 3, the relative activity of the normal sequence (pGL3-SVP230-luc) was significantly higher than that of both the mutated sequence (pGL3-SVP229-luc) and the negative control group in the A549 cells (*p *< 0.01). Another HIF-1α binding site, located at -166 bp~-163 bp of the survivin core promoter, was also mutated, but there was no relative difference in transcriptional activity between the normal and mutated binding site promoter constructs.

### Decreased HIF-1α expression leads to decreased survivin expression in A549 cells

**Figure 4:**
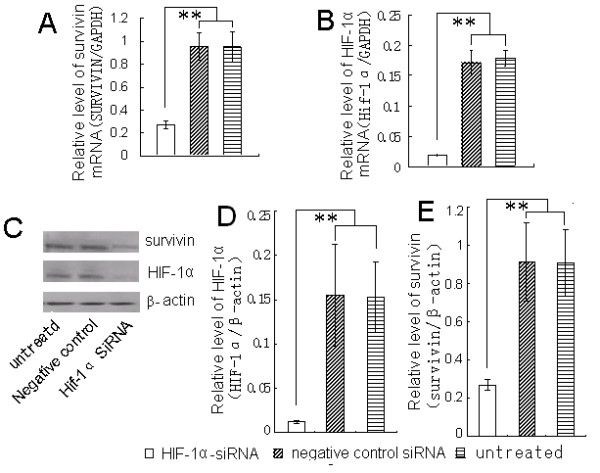
**Decreased HIF-1α expression leads to decreased survivin expression in A549 cells**. Cells were cultured in 10% FBS medium overnight, followed by treatment with HIF-1α-siRNA for 48 h. Total RNAs were isolated and analyzed by quantitative, real time, reverse transcription-PCR to determine the changes of survivin (A) and HIF-1α (B) mRNA. The relative levels of survivin and HIF-1α mRNA are expressed as a ratio of the amount of survivin (A) or HIF-1α (B) PCR products to the amount of GAPDH PCR product. C: The expression of survivin and HIF-1α protein in A549 cells following HIF-1α-siRNA treatment as detected by Western blot analysis. The relative expression levels of HIF-1α (D) and survivin (E) protein is expressed as a ratio of the amount of survivin or HIF-1α protein to the amount of β-actin protein. Data are given as means ± SD, n = 3, ** p < 0.01. Data are given as means ± SD, n = 3, ** p < 0.01.

A549 cells were treated with dsRNA (siRNA) targeted to HIF-1α mRNA and the expression levels of HIF-1α and survivin mRNA, and protein in were detected. As shown in Fig. 4, the mRNA and protein expression levels of HIF-1α and survivin in A549 cells significantly decreased after the treatment with HIF-1α siRNA as compared with negative control siRNA and untreated controls (*p *< 0.05).

## Discussion

Apoptosis has negatively regulates the occurrence and development of tumors and prevents the rapid growth of tumor cells. Apoptosis is co-regulated by apoptosis-promoting factor and apoptosis-inhibiting factors (such as members of the IAP family of proteins) [22,23]. Survivin, the smallest protein of IAP family, is rarely expressed in differentiated tissues and highly express in 75 ~ 96% of tumor tissues [4]. In this study, we found that survivin was expressed in 81.6% of NSCLC tissues, and not expressed in tissues from patients with benign lung diseases. The expression of suvivin in the lung cancer tissue samples was related to the differentiation, lymph node metastasis and clinical stage of the cancer. These data suggest that survivin plays an important role in prompting the development of lung cancer.

In recent years, studies have showed that the activity of survivin promoter in tumor cells is significantly increased [24-27]. This suggests that the expression of survivin is transcriptional regulated. Reduction of promoter activity could significantly decrease the mRNA and thus decrease the protein expression of survivin. Although the survivin promoter contains several GC boxes, but methylation of these GC boxes has not been found in the survivin promoter. It is implicit that the regulation of survivin expression is at the level of transcription but it is still unclear how survivin transcription is regulated by the Cis-acting elements.

HIF-1α is highly expressed in various tumor tissues and plays an important role in regulating hypoxia, and tumor invasion and progress [17,19,20]. In this study, we confirmed that HIF-1α is highly expressed in NSCLC tissue, as was found in breast cancer [28]. The expression of HIF-1α is related to differentiation, lymph node metastasis and clinical stage of lung cancer. Correlation analysis showed the expression of survivin was positively correlated with HIF-1α. The previous studies have showed that HIF-1α is intermediate link in the evolution of the tumor, and this protein could regulate a variety of hypoxia-induced gene expression [29]. In vitro, we also found that the expressions of HIF-1α and survivin in A549 cells were significantly increased under hypoxic conditions. Therefore, we speculated that HIF-1α might be a transcriptional activator of survivin. An early study using bioinformatic analysis of the *survivin *promoter 5'-upstream non-coding region found that the *survivin *gene TSS (transcriptional start site) was located in -64 bp upstream of translation initiation codon (ATG). This bioinformatic analysis also showed that the potential transcription factors that could bind to the survivin promoter included Sp1, E2F, p53, CDE, CHR, etc [14]. Our study detected that there are also 2 putative binding sites for HIF-1α, which are located at-16 bp to -19 bp and at -133 bp to -136 bp in the proximal promoter region of human *survivin *gene. The first site (16 bp to -19 bp) partially overlaps with one of the potential Sp1 binding sites. Peng et al [20] also confirmed that there is a putative HIF-1α binding site in the survivin core promoter (-203 to +27). They also found that in breast cancer cells, HIF-1α, induced by EGF, could bind to this putative binding-site under hypoxic or normoxic conditions and that when HIF-1α is bound to its binding site in the survivin promoter the expression of survivin is increased [20]. In order to investigate if there are similar transcriptional regulation mechanisms in lung cancer, we constructed two reporter constructs; one with the normal putative binding site of HIF-1α (-19 bp ~-16 bp) in the survivin core promoter and another with a mutated form of this binding site. These constructs were then transfected into A549 lung cancer cells. The results showed that the relative activity of the mutation of this HIF-1α binding site reduced transcriptional activity by 36.60%. Another HIF-1α binding site, located at -166 bp~-163 bp of the survivin core promoter was also mutated, but there was no relative difference in transcriptional activity between the normal and mutated binding site promoter constructs (data not show). These data suggest that the site locating at -19 bp ~-16 bp is one of the key cis-acting elements of survivin core promoter.

To further prove that survivin could be induced by HIF-1α, we used RNAi to silence the expression of HIF-1α. Our results showed that the RNAi significantly decreased the expression of HIF-1α mRNA and protein in A549 cells, and that this decrease of HIF-1α correlated with the decreased expression of survivin. This suggests that inhibiting expression of the HIF-1α gene can decrease the expression of survivin, and that HIF-1α might be an important transcription factor involved in the regulation of survivin mRNA expression.

## Conclusion

In summary, our experimental results demonstrated that HIF-1α and survivin are highly expressed in non-small cell lung cancer and lung adenocarcinoma cell line A549 cells, and that the expression of these proteins correlated with one another. Additionally, we show that hypoxia could induce the expression of HIF-1α and survivin. Furthermore, the data presented here demonstrate that the potential binding site of HIF-1α on survivin promoter has a positive role in the regulation of transcriptional activity of the survivin gene, HIF-1α may be an important transcription factor involved in regulation of survivin expression.

## Competing interests

The authors declare that they have no competing interests.

## Authors' contributions

YQC designed the experiments and wrote the manuscript; CLZ and WL carried out the the molecular genetic studies, immunoassays and the statistical analysis. All authors read and approved the final manuscript.
